# Suppression of angiotensin converting enzyme 2, a host receptor for SARS-CoV-2 infection, using 5-aminolevulinic acid *in vitro*

**DOI:** 10.1371/journal.pone.0281399

**Published:** 2023-02-09

**Authors:** Eriko Nara, Hung Wei Lai, Hideo Imazato, Masahiro Ishizuka, Motowo Nakajima, Shun-Ichiro Ogura

**Affiliations:** 1 School of Life Science and Technology, Tokyo Institute of Technology, Midori-ku, Yokohama, Japan; 2 SBI Pharmaceuticals Co. Ltd., Minato-ku, Tokyo, Japan; Osaka Rosai Hospital: Osaka Rosai Byoin, JAPAN

## Abstract

Angiotensin converting enzyme 2 (ACE2), an entry receptor found on the surface of host cells, is believed to be detrimental to the infectious capability of severe acute respiratory syndrome coronavirus 2 (SARS-CoV-2). Scientists have been working on finding a cure since its outbreak with limited success. In this study, we evaluated the potential of 5-aminolevulinic acid hydrochloride (ALA) in suppressing ACE2 expression of host cells. ACE2 expression and the production of intracellular porphyrins following ALA administration were carried out. We observed the reduction of ACE2 expression and intracellular porphyrins following ALA administration. ALA suppressed the ACE2 expression in host cells which might prevent binding of SARS-CoV-2 to host cells. Co-administration of ALA and sodium ferrous citrate (SFC) resulted in a further decrease in ACE2 expression and increase in intracellular heme level. This suggests that the suppression of ACE2 expression by ALA might occur through heme production. We found that the inhibition of heme oxygenase-1 (HO-1), which is involved in heme degradation, also resulted in decrease in ACE2 expression, suggesting a potential role of HO-1 in suppressing ACE2 as well. In conclusion, we speculate that ALA, together with SFC administration, might serve as a potential therapeutic approach in reducing SARS-CoV-2 infectivity through suppression of ACE2 expression.

## Introduction

Incidents of patients with pneumonia of unidentified causes were reported in large numbers in late 2019 [1]. The pathogen responsible was later identified in local hospitals as severe acute respiratory syndrome-corona virus-2 (SARS-CoV-2) [2]. Patients infected by SARS-CoV-2 were named as coronavirus disease 2019 (COVID-19) patients by WHO [3]. As of August 2022, 581,686,197 confirmed cases of COVID-19, including 6,410,961 deaths have been reported globally to WHO [3]. Despite scientists’ continuous efforts around the world, there is still no effective cure for COVID-19 besides employing clinical managing strategies and providing supportive care for severely ill patients [4, 5].

Angiotensin-converting enzyme 2 (ACE2), a receptor found on the surface of human cells, plays a pivotal role in renin-angiotensin-aldosterone system, regulating blood pressure and electrolyte homeostasis [6]. However, the high affinity of viral spike protein of SARS-CoV-2 towards ACE2 has allow viral entry into human cells through S-protein priming by the host cell protease, TMPRSS2 [6]. It is also reported that the binding affinity of S-protein and ACE2 correlate with the replication rate of the virus and severity of the disease [7]. The findings also correlate with the reports where high ACE2 expression leads to higher viral load in nasal swabs and higher disease severity of COVID-19 patients [8, 9]. Interestingly, the study by Bunyavanich *et*. *al*., (2020), showed that children under age 18 have significantly lower ACE2 expression compared to adults, suggesting low SARS-CoV-2 infectivity among children [10]. Therefore, it is believed that the suppression of ACE2 may be a novel method to reduce infectious capability of SARS-CoV-19.

**Fig 1:**
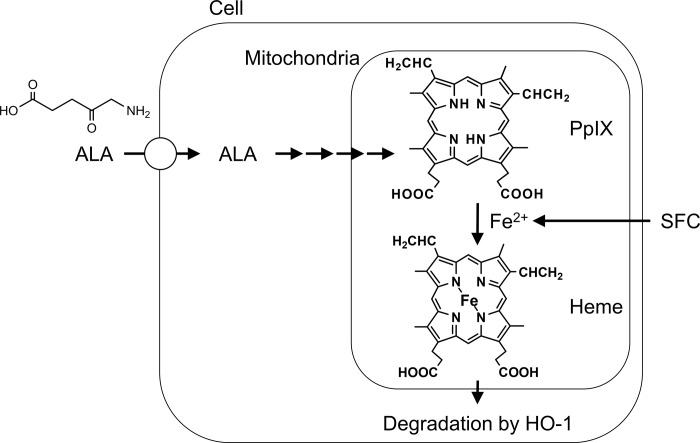
Schematic illustration indicating the uptake of 5-Aminolevulinic acid and conversion of PpIX into heme.

5-Aminolevulinic acid (ALA) is an essential amino acid, with little toxicity, naturally found in the human body [11]. The conjugation of eight molecules of 5-Aminolevulinic acid lead to the formation of protoporphyrin IX (PpIX), which may be converted in heme prior to the insertion of ferrous ion (Fig 1) [12]. Since administration of ALA lead to heme formation, which is essential in maintaining the optimum function of protein complexes such as cytochromes, ALA might be used to improve metabolic processes in various diseases such as diabetes [13]. In addition, ALA has been used in photodynamic diagnosis and photodynamic therapy of various cancers at a very high dose such as 20 mg/kg body weight which might cause some side effects [14–18]. A preliminary study by Sakurai et al. (2021) [19], suggested the reduced infectivity of SARS-CoV-2 following ALA administration, although the molecular mechanisms leading to this phenomenon were not yet evaluated [20]. We hypothesized that ALA might inhibit infectivity of SARS-CoV-2 by suppressing the expression of ACE2 which is a host cell receptor for S-protein of SARS-CoV-2, possibly due to increase in heme production following ALA administration. We studied the mechanism of 5-Aminolevulinic acid in inhibiting SARS-CoV-2 infectivity.

## Methods

### Cells and cell culture

The human liver cancer cell line, HepG2, was provided by SBI Pharmaceuticals Co. Ltd. (Tokyo, Japan). VeroE6, a cultured cell line derived from kidney epithelial cells isolated from African green monkeys, was obtained from JCRB Cell Bank, National Institutes of Biomedical Innovation, Health and Nutrition, Japan. The cells were cultured in DMEM (Low) culture medium containing 10% foetal bovine serum (FBS) and 10% ABAM, at 37°C in a 5% CO_2_ incubator. Experiments were performed at a cell density of 50%–80% confluence.

### Biochemicals

DMEM (Low) culture medium and antibiotic–antimycotic mixed stock medium (ABAM) were purchased from Nacalai Tesque (Kyoto, Japan). FBS was purchased from Equitech-Bi, Inc. (Kerrville, TX, USA). 5-Aminolevulinic acid hydrochloride (ALA) (neo ALA Co. Ltd, Tokyo, Japan) and sodium ferrous citrate (SFC) (Komatsuya Corporation, Osaka, Japan) were provided by SBI Pharmaceuticals Co. Ltd. (Tokyo, Japan). Zinc protoporphyrin IX (ZnPpIX) was purchased from Frontier Scientific (Utah, US).

### Western blot analyses

Western blotting analyses were carried out as previously described [21]. We used monoclonal anti-human ACE2 antibody (Abcam, Cambridge, UK; 1:1000) and anti-human actin antibody (ThermoFisher Scientific, Massachusetts, USA; 1:600 dilution) as primary antibodies. Secondary antibodies were horseradish peroxidase (HRP)-conjugated anti-mouse (Cell Signaling Technology, Beverly, MA, USA) and anti-rabbit IgG (Santa Cruz Biotechnology, Dallas, TX, USA) concentrates, diluted 3,000 times in tris-buffered saline (TBST) solution.

### HPLC analysis of PpIX and heme

Quantification of PpIX and heme by HPLC were carried out by preparing cell lysate through administration of 0.1 M NaOH to PBS-washed samples. A protein denaturant equivalent to 3 times of the cell lysate was added to extract PpIX and heme in the cells. The protein denaturant was a mixture of mobile phase A (1 M ammonium acetate solution containing 12.5% acetonitrile adjusted to pH = 5.2) and mobile phase B (50 mM ammonium acetate solution containing 80% acetonitrile) solution at a ratio of 1:9 (v/v). Centrifugation was performed twice at 10,000×g for 10 min at 4°C to remove denatured proteins, and the supernatant was collected. Protein concentrations were quantified by the Bradford method using Quickstart™ Bradford 1×Dye Reagent (Bio-rad Laboratories Inc., California, USA). For HPLC analysis of PpIX and heme, a Type Prominence system (Shimadzu, Kyoto, Japan) were used. Mobile phase A and mobile phase B were used for elution of porphyrins. The elution program consisted of 10% mobile phase A and 90% mobile phase B at a flow rate of 2 mL/min for 7 minutes. The eluate was measured by absorbance at 404 nm using a spectroscopic detector. 100 μL of sample was injected. Hemin used as a heme standard was purchased from Nacalai Tesque (Kyoto, Japan). Protoporphyrin IX dihydrochloride used as a standard substance for PpIX was purchased from Frontier Scientific, Inc. (UT, USA).

### Statistical analysis

Microsoft Excel 2010 was used for data analysis in this study. A one-way ANOVA (Tukey’s Test) was performed for each data set to identify differences in mean values between treated and non-treated samples; at two levels of significance, p < 0.05 and p < 0.01.

## Results

### Effect of ALA on ACE2 expression in HepG2 and VeroE6 cells

**Fig 2:**
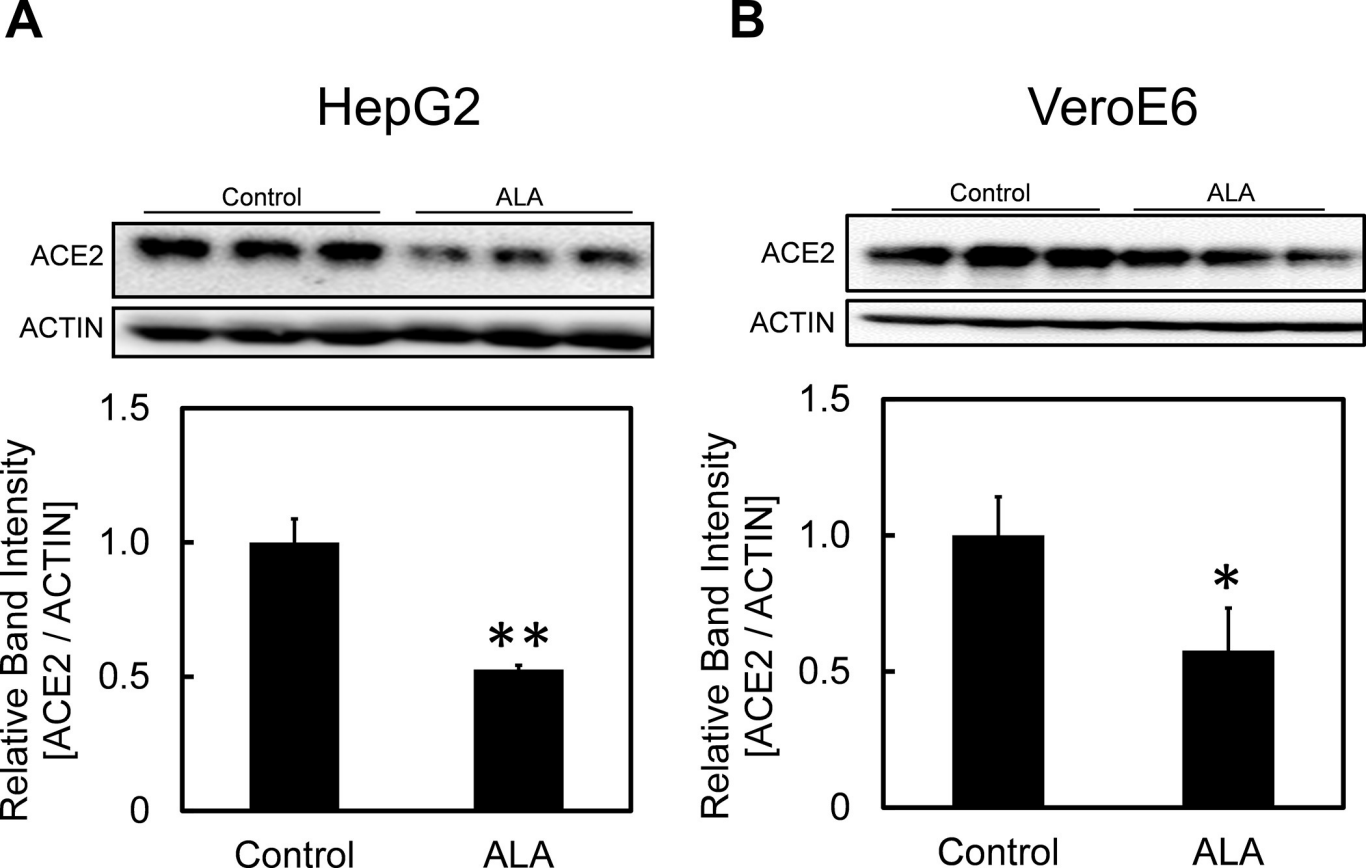
Effect of ALA on ACE2 expression in HepG2 and VeroE6 cells. Changes in protein expression levels of ACE2 in (A) HepG2 and (B) VeroE6 cell lines in the presence or absence of ALA treatment. A one-way ANOVA (Tukey’s test) was performed for each data set to identify differences in mean values between treated and untreated samples; **, p < 0.01; *, p < 0.05. n = 3. Bars represent standard deviation (SD). Blots are cropped to ease visualization. Unprocessed original blot scans are shown in S1 Fig.

In order to study the effect of ALA administration on infectious capability of SARS-CoV-2, we first studied the changes in expression of ACE2, a surface receptor which binds to spike protein of SARS-CoV-2. HepG2 and VeroE6 cells were incubated at 2.15 × 10^4^ cells / cm^2^ at 37°C in 5% CO_2_ -atmosphere for 48 h before evaluating the change in protein expression level of ACE2 using Western blot. Fig 2A showed the expression of ACE2 decreased by approximately 50% following administration of ALA in HepG2 cells. Fig 2B also exhibited lower expression of ACE2 following ALA administration in VeroE6 cells, a cultured cell line commonly used in SARS-CoV-2 study. These results suggested ALA administration led to decrease in availability of ACE2, a surface receptor for SARS-CoV-2, on host cells.

### Changes in intracellular PpIX and heme levels following ALA administration

**Fig 3:**
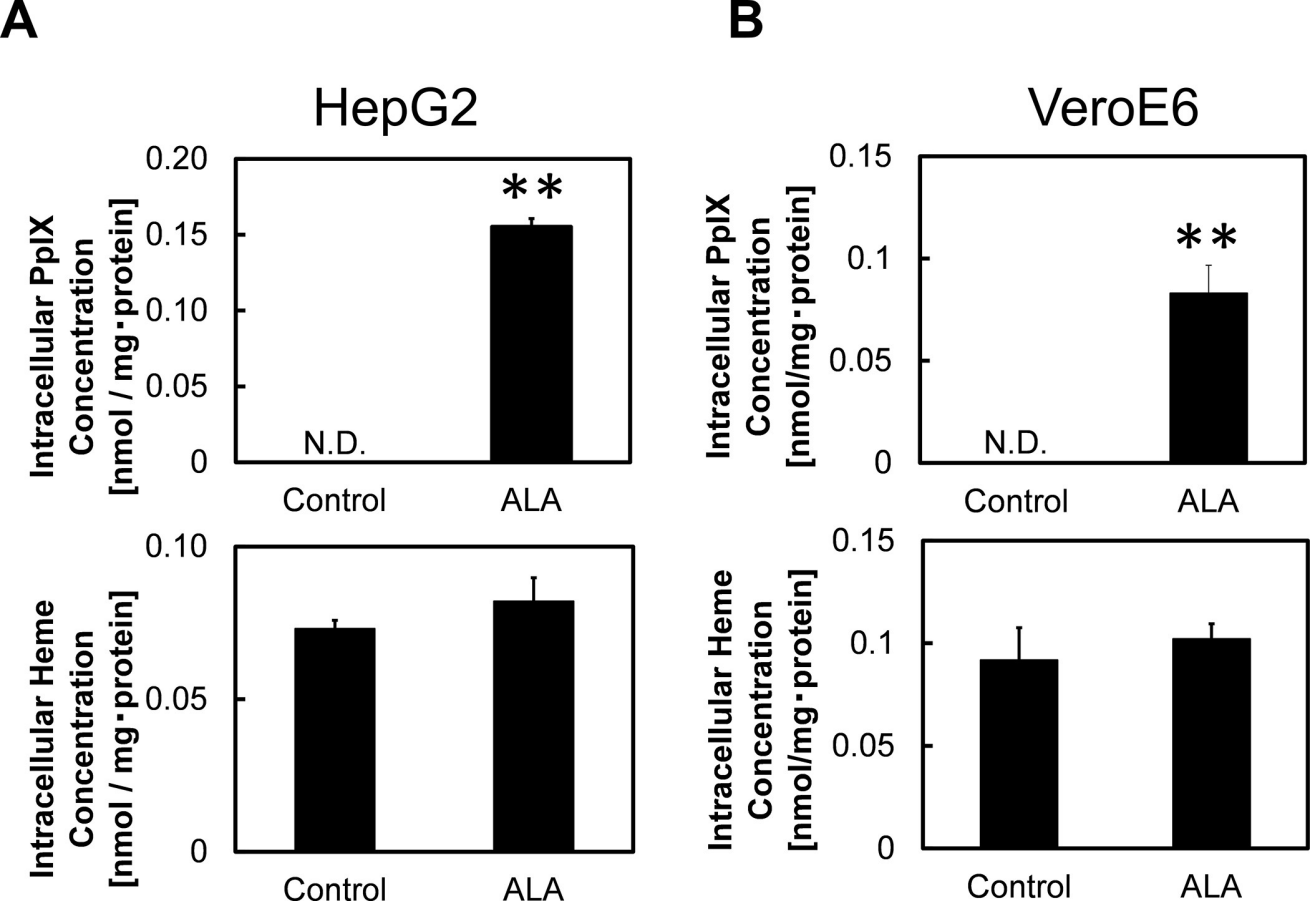
Changes of intracellular PpIX and heme following ALA administration. Concentration of intracellular PpIX and heme in (A) HepG2 cells and (B) VeroE6 cells following administration of ALA. A one-way ANOVA (Tukey’s test) was performed for each data set to identify differences in mean values between treated and untreated samples; **, p < 0.01. n = 3. Bars represent standard deviation (SD).

After its entry into the cell, ALA is converted into PpIX and heme through a series of enzyme-mediated reactions in the cell [22, 23]. The uptake of ALA into the cell can be determined by measuring the concentration of PpIX and heme produced using high performance liquid chromatography (HPLC) as described in the Materials & Methods. We observed significant increases in the concentration of intracellular PpIX in both HepG2 and VeroE6 cell lines (Fig 3). This suggest that ALA was uptaken into the cell and converted into PpIX. However, only a slight increase (statistically insignificant; p > 0.05) in the heme concentration was observed. We hypothesized that this might be due to the lack of iron source which limited the conversion of PpIX into heme.

### Changes in ACE2 expression following ALA and SFC co-administration

**Fig 4:**
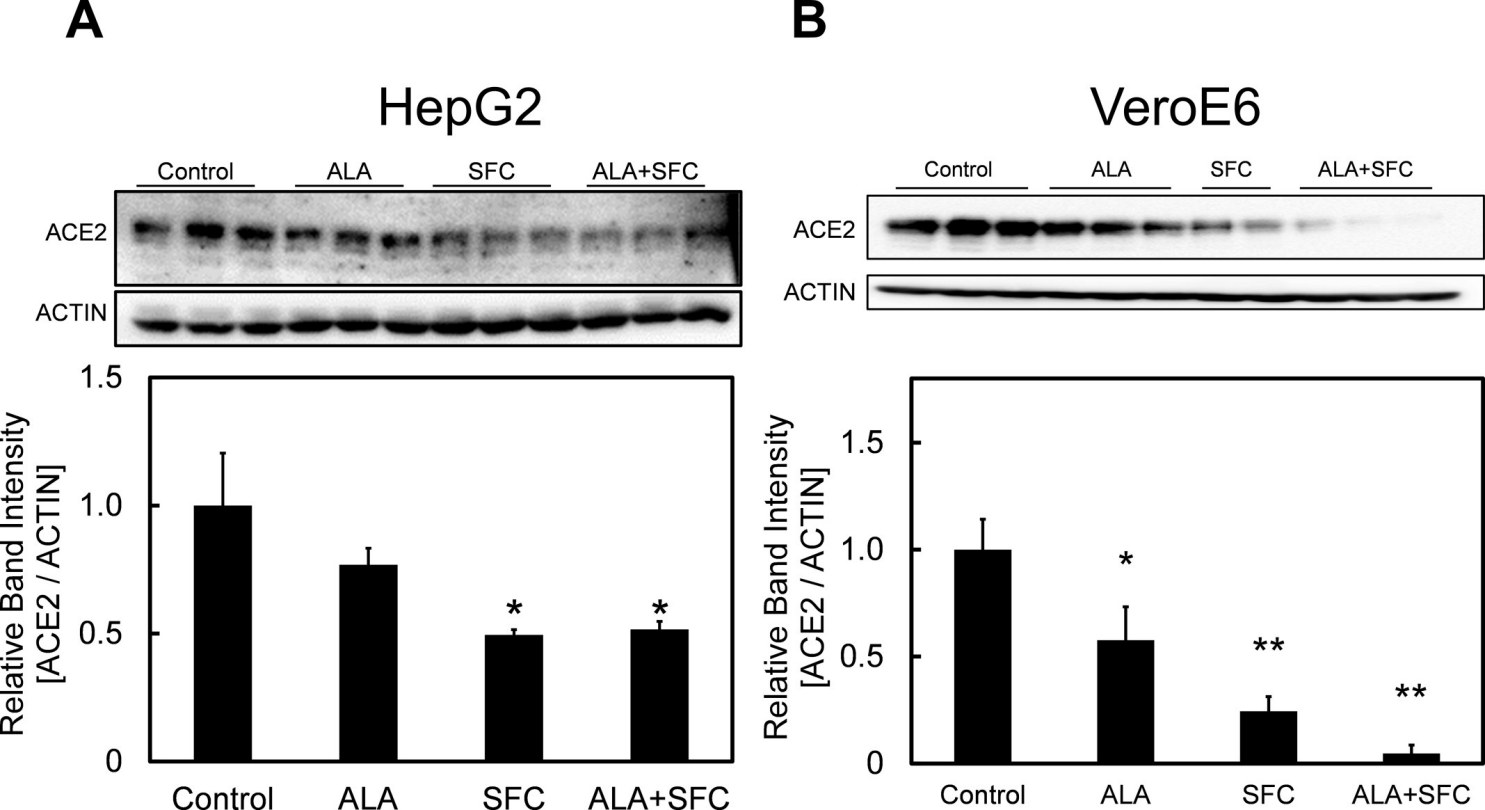
Changes of ACE2 expression following ALA and SFC co-administration. Changes in protein expression levels of ACE2 in (A) HepG2 and (B) VeroE6 cell lines in the presence or absence of ALA and SFC. A one-way ANOVA (Tukey’s test) was performed for each data set to identify differences in mean values between treated and untreated samples; **, p < 0.01; *, p < 0.05. n = 3 or 2. Bars represent standard deviation (SD). Blots are cropped to ease visualization. Unprocessed original blot scans are shown in S2 Fig.

Sodium ferrous citrate (SFC) is commonly used an iron source in various studies [24, 25]. In order to induce more conversion of PpIX into heme, we evaluated the effect of co-administration of ALA and SFC on ACE2 expression. Coincided with results shown in Fig 2, ALA administration resulted in decrease in ACE2 expression (Fig 4). The co-administration of ALA and SFC further reduced the expression levels of ACE2 in both cell lines, particularly in VeroE6 cell line (Fig 4). These findings suggest that SFC may enhance inhibitory effect of ALA on ACE2 expression.

### Changes of porphyrin-heme production and ACE2 suppression following co-administration of ALA and SFC

**Fig 5:**
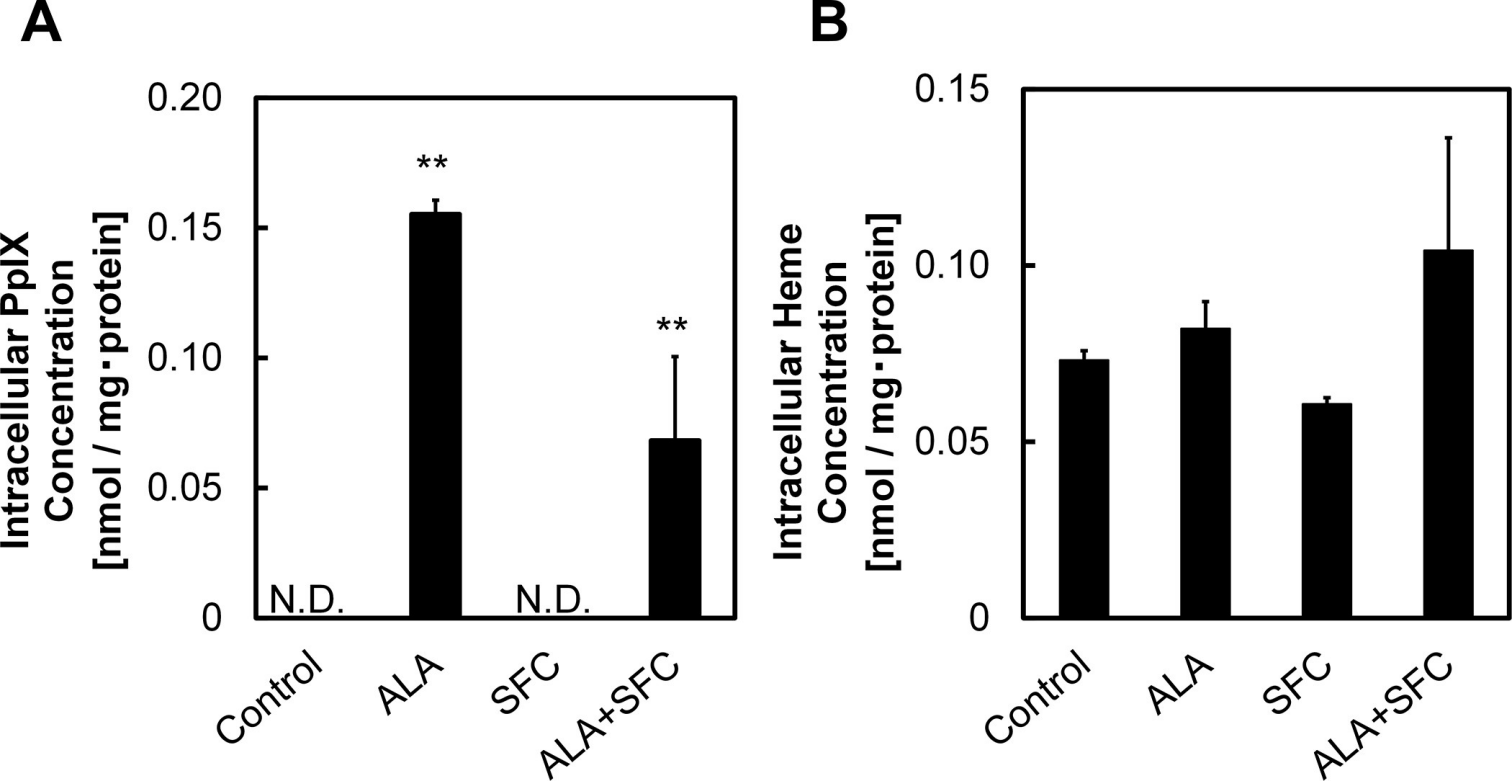
Changes of PpIX and heme production following co-administration of ALA and SFC in VeroE6 cells. Concentration of intracellular (A) PpIX and (B) heme in VeroE6 cells following administration of ALA and SFC. A one-way ANOVA (Tukey’s test) was performed for each data set to identify differences in mean values between treated and untreated samples; **, p < 0.01. n = 3. Bars represent standard deviation (SD). Blots are cropped to ease visualization.

Findings from Fig 4B showed changes of ACE2 expression following the administration of ALA and SFC. In this section, we evaluated the concentration of intracellular PpIX and heme in VeroE6 cells following administration of ALA in the absence and presence of SFC (Fig 5). PpIX level in VeroE6 cells remained undetectable following administration with SFC only. No increase in PpIX production compared to the control was observed (Fig 5A). The co-administration of ALA and SFC resulted in a decrease in PpIX production compared to ALA-treated samples. Fig 5B showed the concentration of heme in VeroE6 cells increased following ALA administration. A slight decrease in heme level was observed following SFC-only treatment. Interestingly, the concentration of intracellular heme increased following co-administration of ALA and SFC. This finding suggests the administration of iron source induce PpIX conversion into heme, leading to increase in ALA-induced heme production and reduction of ACE2 expression.

### Changes of ACE2 expression following ALA administration and HO-1 inhibition

**Fig 6:**
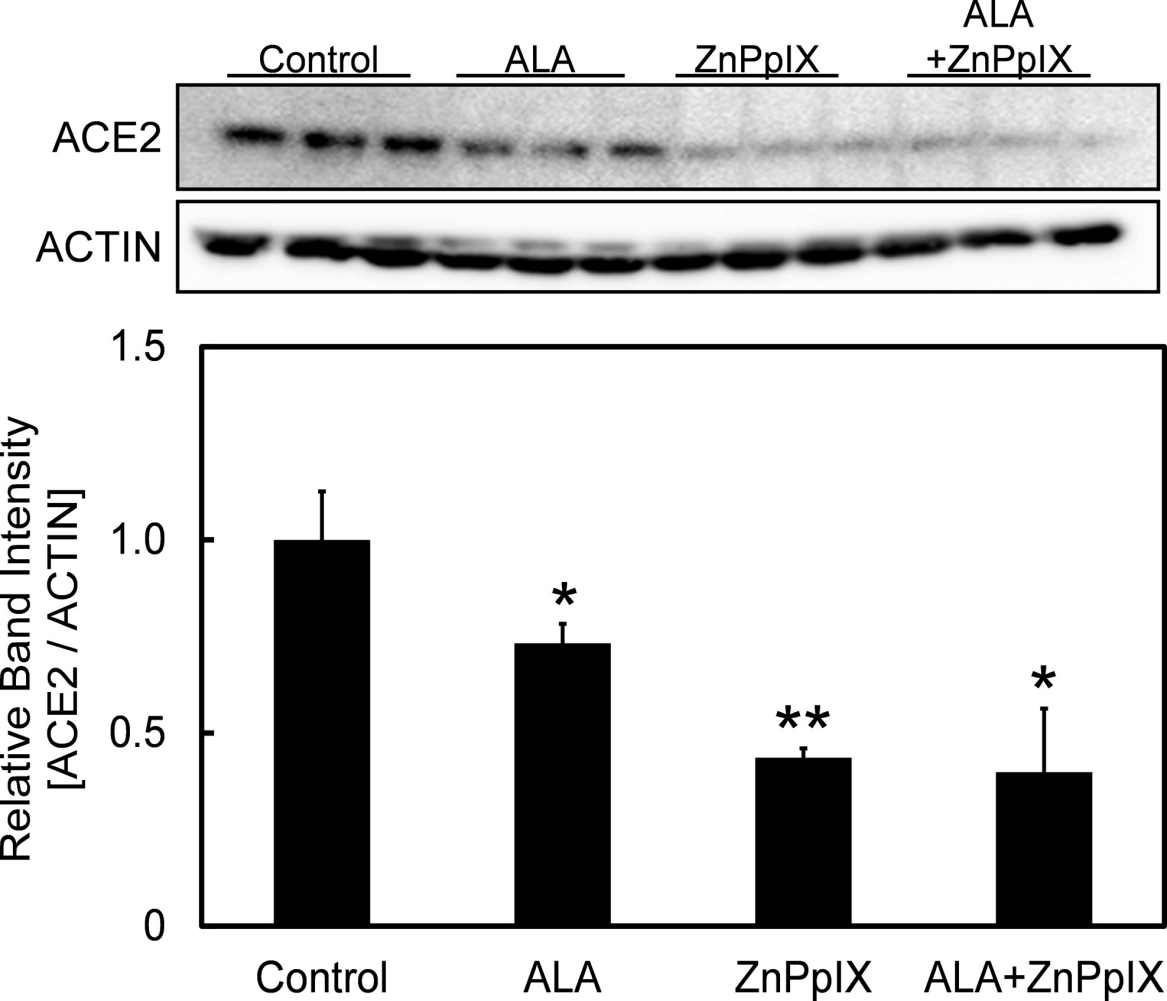
Changes of ACE2 expression following ALA administration and HO-1 inhibition in HepG2 cells. Changes in protein expression levels of ACE2 in HepG2 following administration of ALA and ZnPpIX, a HO-1 inhibitor. A one-way ANOVA (Tukey’s test) was performed for each data set to identify differences in mean values between treated and untreated samples; **, p < 0.01; *, p < 0.05. n = 3. Bars represent standard deviation (SD). Blots are cropped to ease visualization. Unprocessed original blot scans are shown in S3 Fig.

**Fig 7:**
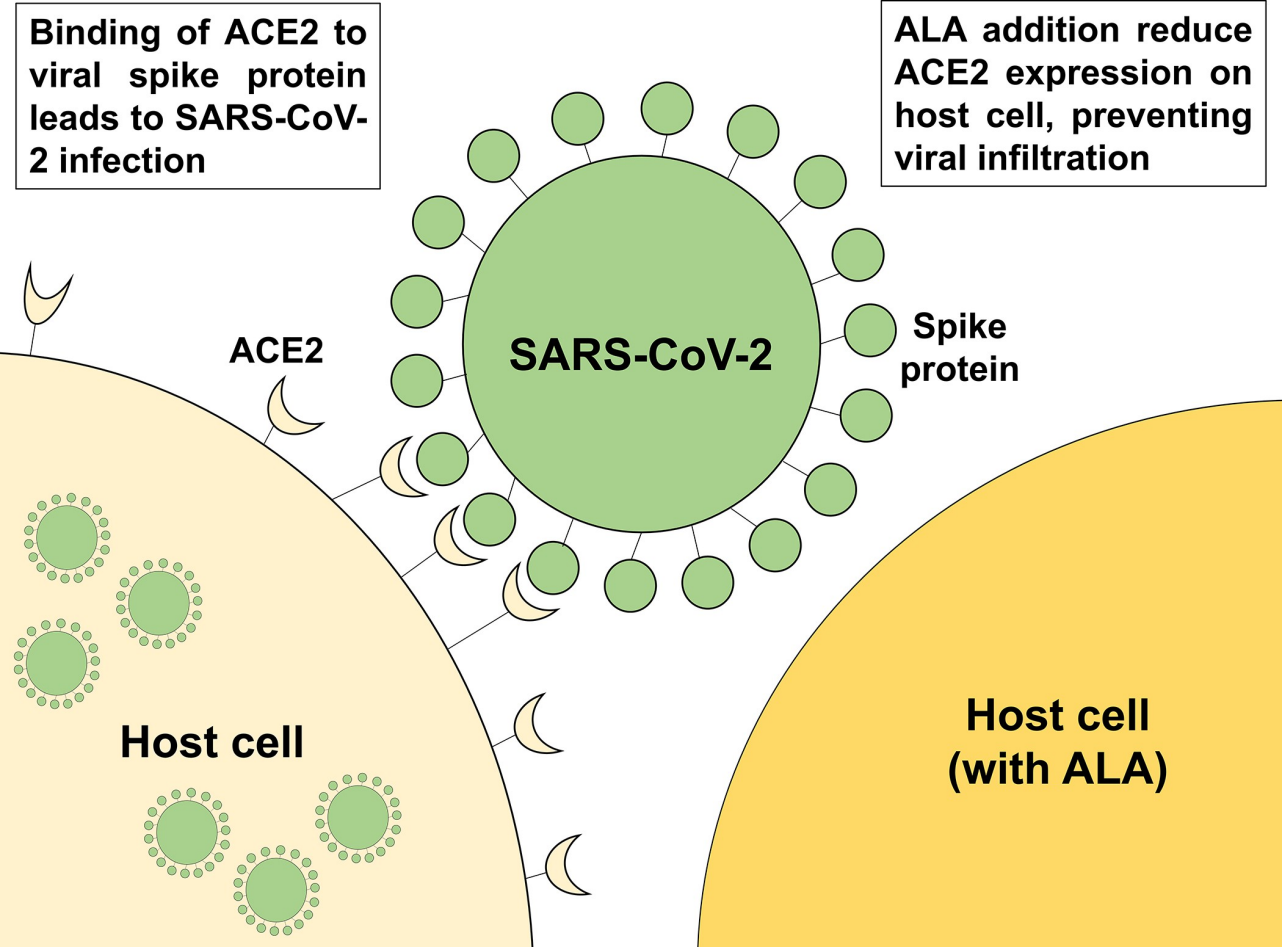
Schematic illustration on the effect SARS-CoV-2 infectivity towards host cell treated with and without ALA. ALA suppressed the ACE2 expression in host cells which might prevent binding of SARS-CoV-2 to host cells.

In this section, we attempted to increase heme production by inhibiting heme oxygenase 1 (HO-1) to evaluate its effect on ACE2 expression. HO-1 is important in catalyzing the degradation of heme into biliverdin and bilirubin [26]. Zinc protoporphyrin IX (ZnPpIX) was used as an inhibitor for suppressing HO-1 activity, leading to high production of heme [27]. Fig 6 showed the changes of protein expression level of ACE2 following ALA and ZnPpIX administration. Coincided with the results shown in Fig 2A, the expression of ACE2 decreased following ALA administration. Moreover, the treatment of HepG2 with ZnPpIX showed a further decrease in ACE2 expression. Our findings suggest that porphyrin and/or heme play a role in inhibiting ACE2 expression, and that the administration of ALA inhibit ACE2 expression through porphyrin and/or heme production (Fig 7).

## Discussion

Aside from clinical managing strategies and supportive care for COVID-19 patients, there is currently no effective cure for treating the disease [4, 5]. The emergence of new SARS-CoV-2 variants which possess higher infectivity and stronger resistance towards currently available vaccines only further worsen the situation [28]. ACE2, a receptor found on the surface of human cells, allows the virus to enter the host cell after binding to viral spike protein [5]. While ACE2 is known to be highly expressed in nasal epithelial cells, it is also reported that the expression of ACE2 increases as people aged [7, 10]. This is particularly dangerous since most elderly people have weaker immune system compared to younger populations [10]. Therefore, it is imperative that scientists need to develop novel drugs (e.g., ACE2-targeting drugs) to fight against SARS-CoV-2.

5-Aminolevulinic acid, a natural amino acid which is highly useful in treating various kinds of diseases through improvement of the body’s metabolic activity, has showed the potential to exert its inhibitory effect on the infectivity of SARS-CoV-2 *in vitro* although the exact mechanism has not yet been evaluated [19, 20]. We hypothesized that ALA possesses its inhibitory effect on ACE2 expression in host cells. Our results shown in Fig 2 demonstrated that the expression of ACE2 was significantly reduced in both cell lines of HepG2 and VeroE6 following ALA administration. The uptake of ALA was shown by the increased of PpIX level (Fig 3) although the level of heme only showed a slight increase (statistically insignificant; p > 0.05) in HepG2 cells. We hypothesized that the increase in heme level might be limited due to the lack of ferrous iron because the insertion of ferrous ion is essential in converting PpIX into heme. Our hypothesis was proven by the observation that the administration of SFC, which acts as an iron source, led to a further decrease in ACE2 expression compared to ALA-only treated samples, particularly in VeroE6 cell line (Fig 4). We believed the addition of SFC affect iron metabolism in the cells which somehow lead to reduction in ACE2 expression, although the exact mechanism remain unclear. This further decrease in ACE2 expression suggest that the effectiveness of ALA in inhibiting infectivity of SARS-CoV-2 might be enhanced by heme production.

It is known that RNA G-quadruplex within transmembrane serine protease 2 and ACE2 of the host cell both play important roles in preventing SARS-CoV-2 entry [29]. In year 2018, Shioda et al. showed PpIX is likely interfere with interaction of G-quadruplex structures [30]. In addition, the Japanese COVID-19 patients who were orally administered with ALA and SFC capsules showed a shorter recovery time than patients which underwent standard care for SARS-CoV-2 infection [31]. We hypothesized the addition of ALA and SFC would in turn generate PpIX production that would interfere with G-quadruplex structures and ACE2, leading to reduced infectivity by SARS-CoV-2. In Fig 5, the roles of PpIX and heme in the expression of ACE2 were determined by evaluating the concentration of intracellular PpIX and heme following ALA and SFC co-administration. The decrease in PpIX level following co-administration of ALA and SFC suggested that some of PpIX were converted into heme molecules in an iron source-abundant environment (Fig 5A). Fig 5B showed that the heme level was elevated following the co-administration of ALA and SFC. In accordance with the finding shown in Fig 5A, the increase in heme level is assumed to be a result of increasing conversion of PpIX to heme following the insertion of ferrous ion.

Kim *et*. *al*. (2021) showed that hemin administration suppressed the replication of SARS-CoV-2 [32]. Hemin is an exogenous source of heme, which is commonly used to treat porphyria-related diseases [33]. Our findings from Figs 4B and 5B suggest the concentration of intracellular heme play an important role in regulating the expression of ACE2. The administration of ALA, which induced an increase in heme level, has been observed to lead to a decrease in the expression of ACE2. In order to prove that ALA administration suppressed SARS-CoV-2 infectivity through elevated heme production, we studied the effect of HO-1 inhibition on ACE2 expression level. HO-1, a heme-degrading enzyme, catalyses the degradation of heme into biliverdin and bilirubin [25]. Inhibition of HO-1 might result in an increase in heme level. As shown in Fig 6, the increase in heme level correlated with the enhancement of the suppressive capability of ALA on the expression of ACE2, suggesting the strong relationship between heme and ACE2 expression. This shows the importance of heme in suppressing ACE2 expression, which prevents binding of viral spike proteins to host cells, and ultimately leads to lowered infectivity of SARS-CoV-2. As an agent which stimulates the porphyrin-heme synthesis pathway and increase heme production, ALA might be a potential drug for preventing infiltration of host cell by SARS-CoV-2 through inhibition of ACE2 expression. In addition, it is important to note that the administration of HO-1 inhibitor, ZnPpIX, showed significant decrease in ACE2 expression (Fig 6), suggesting the expression of HO-1 may also contribute to the change in expression of ACE2 and the infectivity of SARS-CoV-2, although the exact mechanism behind this remain unknown.

In this study, we hypothesized the usage of ALA as a potential anti-viral drug targeting ACE2 for SARS-CoV-2 patients. The nature of ALA administration in human such as the safety is reported based on its application in photodynamic diagnosis for fluorescent guided surgery for tumour resection. Therefore, developing ALA as an anti-viral agent should be realistic plan. On the other hand, ACE2 is known to modulate blood pressure homeostasis in human. Although there were initial concerns regarding the usage of ACE inhibitor as drugs for treating COVID-19 patients, there is currently no scientific evidence which suggests it is potentially harmful to administered patients.

In conclusion, ALA administration to the cells induced higher heme production when iron source was available, resulting in inhibition of ACE2 expression, which could lead to prevention of viral spike protein binding to host cells. However, the direct role of PpIX and/or heme in the inhibition of ACE2 binding to viral spike proteins might be important as well although further studies are required. We suggest ALA as a potential anti-viral agent for SARS-CoV-2 which might play an important role in the eradication of the disease in a global scale in the near future.

## Supporting information

S1 Fig Original blots showing the results from Fig 2A & 2B. Protein expression of ACE2 and Actin in (A) HepG2 and (B) VeroE6 cell lines following ALA administration.
(PDF)

S2 Fig Original blots showing the result from Fig 4A & 4B. Protein expression of ACE2 and Actin in A) HepG2 and (B) VeroE6 cell lines following ALA and SFC co-administration.
(PDF)

S3 Fig Original blots showing the results from Fig 6. Protein expression of ACE2 and Actin in HepG2 cell line following ALA and ZnPpIX administration.
(PDF)

## References

[1] Zhu N, Zhang D, Wang W, Li X, Yang B, Song J, et al. A novel coronavirus from patients with pneumonia in China, 2019. New England journal of medicine. 2020 Jan 24. doi: 10.1056/NEJMoa2001017 31978945 PMC7092803

[2] Li Q, Guan X, Wu P, Wang X, Zhou L, Tong Y, et al. Early transmission dynamics in Wuhan, China, of novel coronavirus–infected pneumonia. New England journal of medicine. 2020 Jan 29. doi: 10.1056/NEJMoa2001316 31995857 PMC7121484

[3] WHO COVID-19 Explorer. Geneva: World Health Organization, 2022. Available online: https://worldhealthorg.shinyapps.io/covid/ (Retrieved on: 8^th^ August 2022).

[4] Vellingiri B, Jayaramayya K, Iyer M, Narayanasamy A, Govindasamy V, Giridharan B, et al. COVID-19: A promising cure for the global panic. Science of the total environment. 2020 Jul 10;725:138277. doi: 10.1016/j.scitotenv.2020.138277 32278175 PMC7128376

[5] Bourgonje AR, Abdulle AE, Timens W, Hillebrands JL, Navis GJ, Gordijn SJ, et al. Angiotensin‐converting enzyme 2 (ACE2), SARS‐CoV‐2 and the pathophysiology of coronavirus disease 2019 (COVID‐19). The Journal of pathology. 2020 Jul;251(3):228–48. doi: 10.1002/path.5471 32418199 PMC7276767

[6] Li W, Moore MJ, Vasilieva N, Sui J, Wong SK, Berne MA, et al. Angiotensin-converting enzyme 2 is a functional receptor for the SARS coronavirus. Nature. 2003 Nov;426(6965):450–4. doi: 10.1038/nature02145 14647384 PMC7095016

[7] Sungnak W, Huang N, Bécavin C, Berg M, Queen R, Litvinukova M, et al. SARS-CoV-2 entry factors are highly expressed in nasal epithelial cells together with innate immune genes. Nature medicine. 2020 May;26(5):681–7. doi: 10.1038/s41591-020-0868-6 32327758 PMC8637938

[8] Zhou P, Yang XL, Wang XG, Hu B, Zhang L, Zhang W, et al. A pneumonia outbreak associated with a new coronavirus of probable bat origin. nature. 2020 Mar;579(7798):270–3. doi: 10.1038/s41586-020-2012-7 32015507 PMC7095418

[9] Hoffmann M, Kleine-Weber H, Schroeder S, Krüger N, Herrler T, Erichsen S, et al. SARS-CoV-2 cell entry depends on ACE2 and TMPRSS2 and is blocked by a clinically proven protease inhibitor. cell. 2020 Apr 16;181(2):271–80. doi: 10.1016/j.cell.2020.02.052 32142651 PMC7102627

[10] Bunyavanich S, Do A, Vicencio A. Nasal gene expression of angiotensin-converting enzyme 2 in children and adults. Jama. 2020 Jun 16;323(23):2427–9. doi: 10.1001/jama.2020.8707 32432657 PMC7240631

[11] Ishizuka M, Abe F, Sano Y, Takahashi K, Inoue K, Nakajima M, et al. Novel development of 5-aminolevurinic acid (ALA) in cancer diagnoses and therapy. Int Immunopharmacol. 2011 Mar 1;11(3):358–65. doi: 10.1016/j.intimp.2010.11.029 21144919

[12] Fujino M, Nishio Y, Ito H, Tanaka T, Li XK. 5-Aminolevulinic acid regulates the inflammatory response and alloimmune reaction. International Immunopharmacology. 2016 Aug 1;37:71–8. doi: 10.1016/j.intimp.2015.11.034 26643355

[13] Rehani PR, Iftikhar H, Nakajima M, Tanaka T, Jabbar Z, Rehani RN. Safety and mode of action of diabetes medications in comparison with 5-aminolevulinic acid (5-ALA). Journal of Diabetes Research. 2019 Nov 6;2019. doi: 10.1155/2019/4267357 31781665 PMC6874935

[14] Koizumi N, Harada Y, Minamikawa T, Tanaka H, Otsuji E, Takamatsu T. Recent advances in photodynamic diagnosis of gastric cancer using 5-aminolevulinic acid. World Journal of Gastroenterology. 2016 Jan 1;22(3):1289. doi: 10.3748/wjg.v22.i3.1289 26811665 PMC4716038

[15] Fukuhara H, Kurabayashi A, Furihata M, Setuda S, Takahashi K, Murakami K, et al. 5-aminolevulinic acid-mediated photodynamic diagnosis using fluorescence ureterorenoscopy for urinary upper tract urothelial carcinoma∼ Preliminary prospective single centre trial∼. Photodiagnosis and Photodynamic Therapy. 2020 Mar 1;29:101617. 31857216 10.1016/j.pdpdt.2019.101617

[16] Suero Molina E, Stögbauer L, Jeibmann A, Warneke N, Stummer W. Validating a new generation filter system for visualizing 5-ALA-induced PpIX fluorescence in malignant glioma surgery: a proof of principle study. Acta Neurochirurgica. 2020 Apr;162(4):785–93. doi: 10.1007/s00701-020-04227-7 32034493 PMC7066295

[17] Inoue K, Anai S, Fujimoto K, Hirao Y, Furuse H, Kai F, et al. Oral 5-aminolevulinic acid mediated photodynamic diagnosis using fluorescence cystoscopy for non-muscle-invasive bladder cancer: a randomized, double-blind, multicentre phase II/III study. Photodiagnosis and photodynamic therapy. 2015 Jun 1;12(2):193–200. doi: 10.1016/j.pdpdt.2015.03.008 25843912

[18] Wachowska M, Muchowicz A, Firczuk M, Gabrysiak M, Winiarska M, Wańczyk M, et al. Aminolevulinic acid (ALA) as a prodrug in photodynamic therapy of cancer. Molecules. 2011 May 19;16(5):4140–64.

[19] Sakurai Y, Tun MM, Kurosaki Y, Sakura T, Inaoka DK, Fujine K, et al. 5-amino levulinic acid inhibits SARS-CoV-2 infection in vitro. Biochemical and Biophysical Research Communications. 2021 Mar 19;545:203–7. 33571909 10.1016/j.bbrc.2021.01.091 PMC7846235

[20] Ngwe Tun MM, Sakura T, Sakurai Y, Kurosaki Y, Inaoka DK, Shioda N, et al. Antiviral activity of 5-aminolevulinic acid against variants of severe acute respiratory syndrome coronavirus 2. Tropical Medicine and Health. 2022 Dec;50(1):1–9. 34991723 10.1186/s41182-021-00397-x PMC8739347

[21] Lai HW, Takahashi K, Nakajima M, Tanaka T, Ogura SI. Efficiency of aminolevulinic acid (ALA)-photodynamic therapy based on ALA uptake transporters in a cell density-dependent malignancy model. Journal of Photochemistry and Photobiology B: Biology. 2021 May 1;218:112191. doi: 10.1016/j.jphotobiol.2021.112191 33862352

[22] Lai HW, Sasaki R, Usuki S, Nakajima M, Tanaka T, Ogura SI. Novel strategy to increase specificity of ALA-Induced PpIX accumulation through inhibition of transporters involved in ALA uptake. Photodiagnosis and Photodynamic Therapy. 2019 Sep 1;27:327–35. doi: 10.1016/j.pdpdt.2019.06.017 31252141

[23] Lai HW, Nakayama T, Ogura SI. Key transporters leading to specific protoporphyrin IX accumulation in cancer cell following administration of aminolevulinic acid in photodynamic therapy/diagnosis. International Journal of Clinical Oncology. 2021 Jan;26(1):26–33. doi: 10.1007/s10147-020-01766-y 32875514

[24] Suprihadi A, Pustimbara A, Ogura SI. 5-aminolevulinic acid and sodium ferrous citrate decreased cell viability of gastric cancer cells by enhanced ROS generation through improving COX activity. Photodiagnosis and Photodynamic Therapy. 2022 Dec 1;40:103055. doi: 10.1016/j.pdpdt.2022.103055 35934181

[25] Shimura M, Nozawa N, Ogawa-Tominaga M, Fushimi T, Tajika M, Ichimoto K, et al. Effects of 5-aminolevulinic acid and sodium ferrous citrate on fibroblasts from individuals with mitochondrial diseases. Scientific reports. 2019 Jul 22;9(1):1–1. 31332208 10.1038/s41598-019-46772-x PMC6646320

[26] Hirai K, Sasahira T, Ohmori H, Fujii K, Kuniyasu H. Inhibition of heme oxygenase‐1 by zinc protoporphyrin IX reduces tumor growth of LL/2 lung cancer in C57BL mice. International Journal of Cancer. 2007 Feb 1;120(3):500–5. doi: 10.1002/ijc.22287 17066448

[27] Maines MD. Heme oxygenase: function, multiplicity, regulatory mechanisms, and clinical applications. The FASEB journal. 1988 Jul;2(10):2557–68. 3290025

[28] Tao K, Tzou PL, Nouhin J, Gupta RK, de Oliveira T, Kosakovsky Pond SL, et al. The biological and clinical significance of emerging SARS-CoV-2 variants. Nature Reviews Genetics. 2021 Dec;22(12):757–73. doi: 10.1038/s41576-021-00408-x 34535792 PMC8447121

[29] Liu G, Du W, Sang X, Tong Q, Wang Y, Chen G, et al. RNA G-quadruplex in TMPRSS2 reduces SARS-CoV-2 infection. Nature communications. 2022 Mar 17;13(1):1–3. 10.1038/s41467-022-29135-5 PMC8931161 35301316

[30] Shioda N, Yabuki Y, Yamaguchi K, Onozato M, Li Y, Kurosawa K, et al. Targeting G-quadruplex DNA as cognitive function therapy for ATR-X syndrome. Nature medicine. 2018 Jun;24(6):802–13. doi: 10.1038/s41591-018-0018-6 29785027

[31] Imamura K, Sugihara H, Hirahata K. Phase 2 randomized clinical trial of 5-Aminolevulinic acid plus sodium citrate chloride vs placebo for Covid-19 infected patients recovered with sequelae. ALA-Porphyrin Science. 2022 Mar; 10(1):15–22.

[32] Kim DH, Ahn HS, Go HJ, Kim DY, Kim JH, Lee JB, et al. Hemin as a novel candidate for treating COVID-19 via heme oxygenase-1 induction. Scientific reports. 2021 Nov 2;11(1):1–9. 34728736 10.1038/s41598-021-01054-3 PMC8563742

[33] Yamamoto S, Hara Y, Tomochika KI, Shinoda S. Utilization of hemin and hemoglobin as iron sources by Vibrio parahaemolyticus and identification of an iron-repressible hemin-binding protein. FEMS microbiology letters. 1995 May 1;128(2):195–200. doi: 10.1111/j.1574-6968.1995.tb07522.x 7750738

