# Supplementary material for: Suppression of angiotensin converting enzyme 2, a host receptor for SARS-CoV-2 infection, using 5-aminolevulinic acid in vitro
Source: PLoS One. 2023 Feb 9;18(2):e0281399. doi: 10.1371/journal.pone.0281399 (PMC9910746; doi:10.1371/journal.pone.0281399)
Supplement: S2 Fig — Protein expression of ACE2 and Actin in A) HepG2 and (B) VeroE6 cell lines following ALA and SFC co-administration. (PDF) [file pone.0281399.s002.pdf]

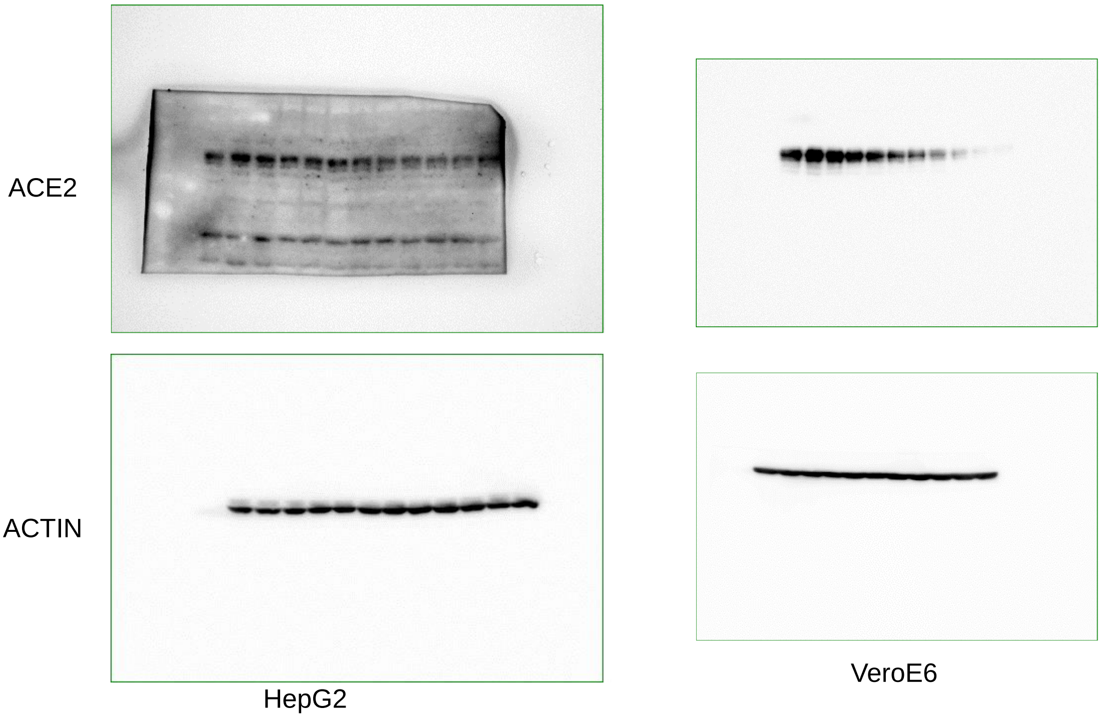

Supplementary Fig. 2. Original blots showing the result from Fig. 4A & 4B. Protein expression of ACE2 and Actin in A) HepG2 and (B) VeroE6 cell lines following ALA and SFC co-administration.
