# Supplementary material for: Suppression of angiotensin converting enzyme 2, a host receptor for SARS-CoV-2 infection, using 5-aminolevulinic acid in vitro
Source: PLoS One. 2023 Feb 9;18(2):e0281399. doi: 10.1371/journal.pone.0281399 (PMC9910746; doi:10.1371/journal.pone.0281399)
Supplement: S3 Fig — Protein expression of ACE2 and Actin in HepG2 cell line following ALA and ZnPpIX administration. (PDF) [file pone.0281399.s003.pdf]

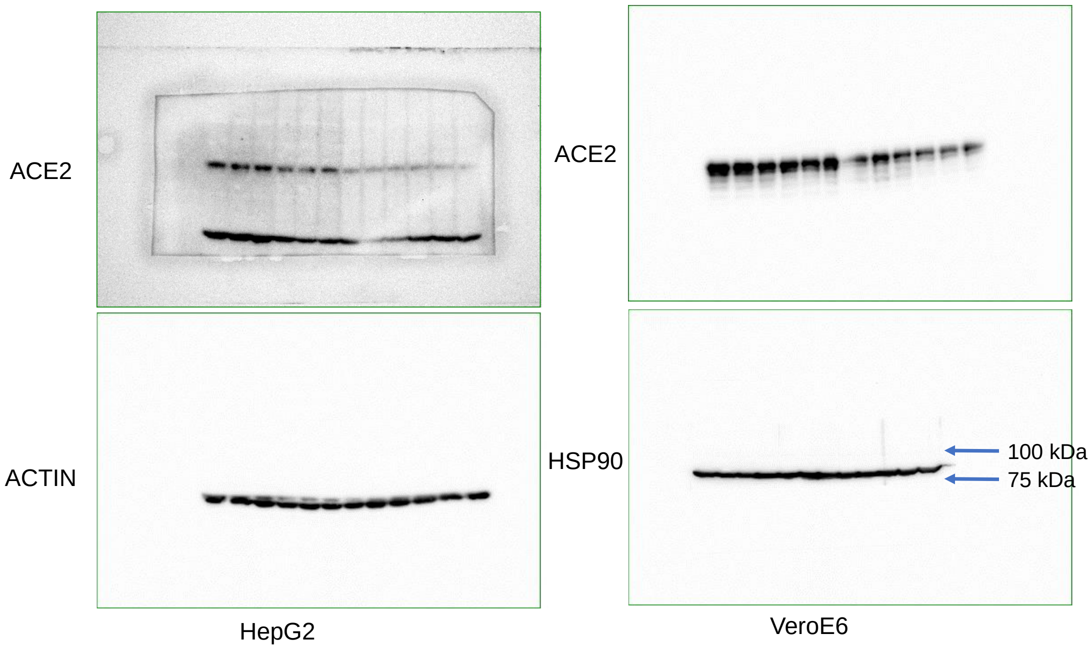

Supplementary Fig. 3. Original blots showing the results from Fig. 6. Protein expression of ACE2 and Actin in HepG2 cell line following ALA and ZnPpIX administration.
